# Endurance Capacities in Professional Soccer Players: Are Performance Profiles Position Specific?

**DOI:** 10.3389/fspor.2020.549897

**Published:** 2020-09-18

**Authors:** Stefan Altmann, Rainer Neumann, Alexander Woll, Sascha Härtel

**Affiliations:** ^1^Department for Performance Analysis, Institute of Sports and Sports Science, Karlsruhe Institute of Technology, Karlsruhe, Germany; ^2^TSG ResearchLab gGmbH, Zuzenhausen, Germany; ^3^Institute of Movement and Sport, University of Education Karlsruhe, Karlsruhe, Germany; ^4^Department for Social and Health Sciences in Sport, Institute of Sports and Sports Science, Karlsruhe Institute of Technology, Karlsruhe, Germany; ^5^TSG 1899 Hoffenheim, Zuzenhausen, Germany

**Keywords:** performance testing, endurance, team sport, football (soccer), match analysis, anaerobic threshold, position specificity

## Abstract

The aim of this study was to investigate position-specific endurance performance of soccer players. 136 professional players competing in the 1st and 2nd division in Germany were divided into the positional groups goalkeepers (GK), central defenders (CD), wingers (WI), central midfielders (CM), and forwards (FW). All players performed an incremental treadmill test with blood lactate sampling until exhaustion with the following endurance parameters being obtained: Fixed aerobic threshold (v_2mmol/l_), fixed anaerobic threshold (v_4mmol/l_), individual aerobic threshold (v_LT_), individual anaerobic threshold (v_IAT_), and maximum velocity (v_max_). Results revealed significant differences between GK and all outfield playing positions for all endurance parameters (*p* ≤ 0.03; ES 0.87–2.19). No significant differences among outfield playing positions were evident for any of the parameters. However, trends were found in favor of the CM compared to the WI (*p* = 0.11; ES = 0.68) and the FW (*p* = 0.06; ES = 0.47) relating to v_LT_ as well as in favor of the CM compared to the WI (*p* = 0.10; ES = 0.56) relating to v_IAT_. Findings suggest that goalkeepers possess the lowest endurance capacity compared to other playing positions. While outfield players in general showed similar endurance performance, CM seem to possess the highest aerobic capacity of all positions as indicated by all lactate-based thresholds, however, with only small to moderate ES. These findings could lead one to question the appropriateness of current endurance training regimes to prepare all players adequately for their positional match-running demands. Indeed, endurance training of players should be specific to their match-running demands. However, it remains unknown to what extent these demands are position or player specific.

## Introduction

Soccer is a team sport characterized by intermittent bouts of intense activity. Per match, professional players cover total distances between 10 and 13 km, while the average running intensity is close to the anaerobic threshold (commonly defined as the running velocity at a blood lactate concentration of 4 mmol/l) (Stølen et al., 2005; Sarmento et al., 2014). Out of this distance, 22–24% is spent at higher intensities (i.e., above 15 km/h), leading to a complex interaction of the aerobic and anaerobic energy systems (Dolci et al., 2020). Moreover, research has shown that the running demands differ between playing positions. More specifically, central midfielders, wide defenders, and wide midfielders cover both the greatest total and high-intensity distance, followed by strikers and central defenders (Di Salvo et al., 2007; Bush et al., 2015). Unsurprisingly, regardless of running intensity, lowest distances have been consistently reported for goalkeepers (West, 2018).

According to the complex demands placed upon the players' endurance capacities, monitoring cardiorespiratory fitness is a well-established component of performance assessments in soccer. In this regard, incremental treadmill tests with simultaneous heart rate and blood lactate sampling are considered a gold-standard method (Impellizzeri et al., 2005). Based on lactate threshold concepts, such tests provide detailed insights into the endurance capacities of the players (Faude et al., 2009).

Given the position-specific aerobic and anaerobic endurance demands during matches, it seems plausible that the players' endurance capacity varies according to their position. To shed light on this issue, Schwesig et al. (2019) analyzed the endurance capacities of German soccer players (3rd and 4th division) using different lactate thresholds derived from an incremental treadmill test. The authors found significant differences in endurance capacities only between goalkeepers and outfield players, while no significant differences were reported among the latter (divided into central defenders, central midfielders, wingers, and forwards). A limitation of this study is that the players were tested at the beginning of the pre-season, where peak performance is likely not reached. Moreover, performance level might be biased by how strictly the individual players adhered to the off-season training plans. Therefore, this point of time might not be optimal to investigate if position-specific levels of endurance performance exist. As mentioned before, the study by Schwesig et al. (2019) analyzed 3rd and 4th division teams from Germany. To date, there is no data available reporting such results for the highest German playing levels (i.e., 1st and 2nd division).

Therefore, the aim of this study was to analyze position-specific endurance performance of soccer players competing in the 1st and 2nd division in Germany utilizing lactate-based thresholds by means of an incremental treadmill test. Data were derived either at the beginning of the first half or the second half of the competitive season, where performance is usually at a high level (Castagna et al., 2013; Manzi et al., 2013). The results of this study could reveal if players of different positions are adequately prepared for their positional running demands during matches and aid coaches to prescribe individual training regimes.

## Materials and Methods

### Participants

A total of 136 male professional soccer players from three clubs competing in the 1st and 2nd German division (1st division *n* = 35; 2nd division *n* = 101; age, 24.5 ± 4.0 years; height, 182.5 ± 6.5 cm; mass, 79.6 ± 7.4 kg) participated. The study was exempt from full ethics review by the institutional review board as the tests were conducted as part of the teams' regular fitness assessments (Winter and Maughan, 2009). All subjects gave written informed consent prior to participation.

### Procedures

All players performed a laboratory-based incremental test on a Woodway treadmill (Woodway GmbH, Weil am Rhein, Germany) with a slope of 1%. Players started at a running speed of 6 km/h which was increased by 2 km/h every 3 min. After each stage, there was a break of 30 s in which capillary blood was collected from the earlobe. Heart rate was monitored using a Polar system (Polar Electro Oy, Kempele, Finland) throughout the whole test. Athletes were instructed to complete as many stages as possible, and the test was finished at volitional exhaustion which was determined by a combination of near-to-maximal values for heart rate, lactate concentrations, and ratings of perceived exertion. Blood lactate concentration for each stage was analyzed utilizing Biosen C-Line Sport (EKF-diagnostic GmbH, Barleben, Germany).

### Data Analysis

To provide comprehensive insights into the endurance capacities of players, a number of parameters were determined by Ergonizer Software (K. Roecker, Freiburg, Germany) according to Faude et al. (2009).

Fixed aerobic threshold (v_2mmol/l_): Velocity at a blood lactate concentration of 2 mmol/l

Fixed anaerobic threshold (v_4mmol/l_): Velocity at a blood lactate concentration of 4 mmol/l

Individual aerobic threshold (v_LT_): Velocity at which blood lactate concentration begins to rise above baseline levels

Individual anaerobic threshold (v_IAT_): Velocity at v_LT_ + blood lactate concentration of 1.5 mmol/l

Maximum velocity (v_max_): Velocity at the point of volitional exhaustion.

The individual thresholds were calculated in addition to the fixed thresholds, as the latter are influenced by initial blood lactate concentrations which differ between player types (e.g., endurance-oriented players vs. speed-oriented players). In addition, maximum velocity was determined as a measure of complex endurance, covering both aerobic (below the anaerobic threshold) and anaerobic (above the anaerobic threshold) components.

In case a player was tested more than one time (the tests were carried out over a 3-year period), all parameters were derived from the test where the highest maximum velocity was reached. To ensure adequate levels of endurance performance, only tests performed either at the beginning of the first half or the second half of the competitive season, respectively, were considered.

As there were no systematic differences in endurance performance between the players competing in the 1st and 2nd division, all data were analyzed for the whole sample. The playing positions were determined according to Schwesig et al. (2019): Goalkeepers, central defenders, wingers, central midfielders, and forwards. No further distinctions between playing positions were made (e.g., dividing wingers into wide defenders and wide midfielders). As running demands during matches can be affected by the tactical system (Baptista et al., 2019), and these systems differed within and between the teams the players belonged to, a further distinction between playing positions was not possible.

### Statistical Analysis

Statistical analysis were performed using SPSS statistical software version 26.0 (SPSS, Inc., Chicago, IL). Mean values and standard deviations (SD) were calculated for the whole sample and the positional groups. One-way analysis of variance (ANOVA) and subsequent Bonferroni *post-hoc* comparisons were used to determine differences in endurance performance between the five positional groups. In addition, Cohen's d effect sizes (ES) were determined to quantify the magnitude of differences between the positions: 0.2 ≤ ES < 0.5 was considered a small effect; 0.5 ≤ ES < 0.8 was considered a moderate effect; ES ≥ 0.8 was considered a large effect (Cohen, 1988). For all statistical tests, the significance level was set a priori to 0.05.

## Results

Descriptive statistics of endurance performance (mean values and SD) separated by playing position are presented in Table 1. Mean differences, ANOVAs, *post-hoc* comparisons, and associated ES between playing positions for the investigated endurance parameters can be found in Tables 2–4. Significant differences were found between goalkeepers and all outfield playing positions for all endurance parameters (*p* ≤ 0.03; ES 0.87–2.19). There were no significant differences among outfield playing positions for any of the parameters. However, trends were found in favor of the central midfielders compared to the wingers (*p* = 0.11; ES = 0.68) and the forwards (*p* = 0.06; ES = 0.47) regarding v_LT_ as well as in favor of the central midfielders compared to the wingers (*p* = 0.10; ES = 0.56) regarding v_IAT_.

**Table 1:** Descriptive results for v_LT_, v_2mmol/l_, v_IAT_, v_4mmol/l_, and v_max_ separated by playing position. Results are presented as mean values ± SD.

	**v_**LT**_ [km/h]**	**v_**2mmol/l**_ [km/h]**	**v_**IAT**_ [km/h]**	**v_**4mmol/l**_ [km/h]**	**v_**max**_ [km/h]**
Whole sample (*n* = 136)	9.3 ± 0.7	12.1 ± 1.5	13.4 ± 1.0	14.7 ± 1.2	17.8 ± 1.0
GK (*n* = 13)	8.3 ± 0.8	10.7 ± 1.2	12.0 ± 0.9	13.2 ± 0.9	16.4 ± 0.8
CD (*n* = 19)	9.4 ± 0.5	12.4 ± 1.2	13.6 ± 0.8	15.0 ± 1.0	17.7 ± 1.0
WI (*n* = 43)	9.3 ± 0.6	12.0 ± 1.6	13.3 ± 0.9	14.7 ± 1.1	18.0 ± 0.9
CM (*n* = 37)	9.7 ± 0.6	12.6 ± 1.5	13.8 ± 0.9	15.2 ± 1.1	18.1 ± 1.0
FW (*n* = 24)	9.2 ± 0.6	12.3 ± 1.2	13.4 ± 0.8	14.8 ± 0.9	17.9 ± 0.8

*SD, Standard deviation; GK, Goalkeepers; CD, Central defenders; WI, Wingers; CM, Central midfielders; FW, Forwards*.

**Table 2:** Mean difference, ANOVA, *post-hoc* test, and ES for fixed thresholds (v_2mmol/l_ and v_4mmol/l_) between playing positions.

	**Mean difference (95% CI) [km/h]**	**ANOVA**	***post-hoc* test**	**ES**
**v**_**2mmol/l**_				
GK vs. CD	−1.8 (−3.2 to −0.3)	*p* < 0.01	*p* < 0.01	1.47
GK vs. WI	−1.4 (−2.6 to −0.1)		*p* = 0.03	0.87
GK vs. CM	−1.9 (−3.2 to −0.7)		*p* < 0.01	1.36
GK vs. FW	−1.7 (−3.0 to −0.3)		*p* < 0.01	1.38
CD vs. WI	0.4 (−0.7–1.5)		*p* > 0.99	0.27
CD vs. CM	−0.2 (−1.3–1.0)		*p* > 0.99	0.14
CD vs. FW	0.1 (−1.1–1.4)		*p* > 0.99	0.09
WI vs. CM	−0.6 (−1.5–0.3)		*p* = 0.68	0.39
WI vs. FW	−0.3 (−1.3–0.7)		*p* > 0.99	0.21
CM vs. FW	0.3 (−0.8–1.3)		*p* > 0.99	0.22
**v**_**4mmol/l**_				
GK vs. CD	−1.8 (−2.8 to −0.7)	*p* < 0.01	*p* < 0.01	1.94
GK vs. WI	−1.5 (−2.4 to −0.6)		*p* < 0.01	1.44
GK vs. CM	−2.0 (−2.9 to −1.0)		*p* < 0.01	1.82
GK vs. FW	−1.6 (−2.6 to −0.6)		*p* < 0.01	1.83
CD vs. WI	0.3 (−0.6–1.1)		*p* > 0.99	0.28
CD vs. CM	−0.2 (−1.0–0.7)		*p* > 0.99	0.19
CD vs. FW	0.2 (−0.8–1.1)		*p* > 0.99	0.22
WI vs. CM	−0.5 (−1.1–0.2)		*p* = 0.53	0.46
WI vs. FW	−0.1 (−0.9–0.6)		*p* > 0.99	0.10
CM vs. FW	0.3 (−0.4–1.1)		*p* > 0.99	0.40

*ANOVA, Analysis of variance; ES, Effect size; 95% CI, 95% Confidence interval; GK, Goalkeepers; CD, Central defenders; WI, Wingers; CM, Central midfielders; FW, Forwards*.

**Table 3:** Mean difference, ANOVA, *post-hoc* test, and ES for individual thresholds (v_LT_ and v_IAT_) between playing positions.

	**Mean difference (95% CI) [km/h]**	**ANOVA**	***post-hoc* test**	**ES**
**v**_**LT**_				
GK vs. CD	−1.1 (−1.8 to −0.5)	*p* < 0.01	*p* < 0.01	1.78
GK vs. WI	−1.0 (−1.6 to −0.5)		*p* < 0.01	1.58
GK vs. CM	−1.4 (−1.9 to −0.8)		*p* < 0.01	2.19
GK vs. FW	−0.9 (−1.5 to −0.3)		*p* < 0.01	1.37
CD vs. WI	0.1 (−0.4–0.6)		*p* > 0.99	0.18
CD vs. CM	−0.3 (−0.8–0.2)		*p* > 0.99	0.54
CD vs. FW	0.2 (−0.4–0.7)		*p* > 0.99	0.37
WI vs. CM	−0.4 (−0.7–0.0)		*p* = 0.11	0.68
WI vs. FW	0.1 (−0.4–0.5)		*p* > 0.99	0.17
CM vs. FW	0.5 (−0.1–0.9)		*p* = 0.06	0.47
**v**_**IAT**_				
GK vs. CD	−1.6 (−2.5 to −0.7)	*p* < 0.01	*p* < 0.01	1.97
GK vs. WI	−1.3 (−2.1 to −0.5)		*p* < 0.01	1.47
GK vs. CM	−1.8 (−2.6 to −1.0)		*p* < 0.01	2.04
GK vs. FW	−1.4 (−2.3 to −0.5)		*p* < 0.01	1.73
CD vs. WI	0.2 (−0.5–0.9)		*p* > 0.99	0.35
CD vs. CM	−0.3 (−1.0–0.4)		*p* > 0.99	0.23
CD vs. FW	0.1 (−0.6–0.9)		*p* > 0.99	0.26
WI vs. CM	−0.5 (−1.1–0.1)		*p* = 0.10	0.56
WI vs. FW	−0.1 (−0.7–0.6)		*p* > 0.99	0.12
CM vs. FW	0.4 (−0.2–1.1)		*p* = 0.66	0.47

*ANOVA, Analysis of variance; ES, Effect size; 95% CI, 95% Confidence interval; GK, Goalkeepers; CD, Central defenders; WI, Wingers; CM, Central midfielders; FW, Forwards*.

**Table 4:** Mean difference, ANOVA, *post-hoc* test, and ES for maximum velocity (v_max_) between playing positions.

	**Mean difference (95% CI) [km/h]**	**ANOVA**	***post-hoc* test**	**ES**
**v**_**max**_				
GK vs. CD	−1.3 (−2.3 to −0.4)	*p* < 0.01	*p* < 0.01	1.46
GK vs. WI	−1.6 (−2.4 to −0.8)		*p* < 0.01	1.85
GK vs. CM	−1.7 (−2.6 to −0.9)		*p* < 0.01	1.82
GK vs. FW	−1.5 (−2.4 to −0.6)		*p* < 0.01	1.93
CD vs. WI	−0.2 (−0.9–0.5)		*p* > 0.99	0.33
CD vs. CM	−0.4 (−1.1–0.3)		*p* > 0.99	0.41
CD vs. FW	−0.2 (−1.0–0.6)		*p* > 0.99	0.23
WI vs. CM	−0.2 (−0.8–0.4)		*p* > 0.99	0.11
WI vs. FW	0.1 (−0.6–0.7)		*p* > 0.99	0.12
CM vs. FW	0.3 (−0.4–0.9)		*p* > 0.99	0.22

*ANOVA, Analysis of variance; ES, Effect size; 95% CI, 95% Confidence interval; GK, Goalkeepers; CD, Central defenders; WI, Wingers; CM, Central midfielders; FW, Forwards*.

## Discussion

The purpose of this study was to investigate position-specific endurance performance of soccer players competing in the 1st and 2nd division in Germany utilizing lactate-based thresholds by means of an incremental treadmill test.

Our results revealed that goalkeepers possess the lowest endurance capacity of all playing positions, indicated by large ES of up to 2.19. Conversely, no significant differences were observed between the outfield playing positions in any of the endurance parameters in question. Nevertheless, central midfielders achieved the highest values regarding all parameters, yielding small to moderate ES of 0.47–0.68 compared to wingers and forwards regarding v_LT_ and v_IAT_.

These findings are well in line with recent results of players competing in the 3rd and 4th German division. More specifically, Schwesig et al. (2019) reported goalkeepers to achieve the lowest running velocity at a lactate concentration of 2, 4, and 6 mmol/l. In addition, while no significant differences were evident regarding the outfield positions, central midfielders showed the highest average performance for all lactate concentrations. While further studies investigating positional differences in endurance performance of soccer players have been published, direct comparisons are not possible due to different endurance tests and classifications of positional groups being applied (Sporis et al., 2009; Boone et al., 2012). The same applies to other studies examining endurance capacities of soccer players, which, however, used different procedures as well as age categories and/or did not investigate different playing positions (e.g., McMillan et al., 2005; Ziogas et al., 2011; Hoppe et al., 2013).

The findings of the current study can only partly be explained by the match demands of different positions. Not surprisingly, the running demands placed upon goalkeepers during matches are low (West, 2018), which is reflected by their weak endurance performance in comparison with other positions. Conversely, the endurance capacity of central defenders does not reflect their match-running demands. While match analyses have consistently shown that central defenders cover the lowest total distance and high-intensity distance (Di Salvo et al., 2007; Bush et al., 2015), players of this position possess endurance capacities comparable to the other outfield positions. Also, the superior endurance capacity of central midfielders in comparison with forwards and wingers in the present study does only in parts reflect match-running demands. Indeed, total distance and high-intensity distance during matches of central midfielders are higher compared to forwards. However, running demands for central midfielders are similar for wingers and therefore the differences between central midfielders and wingers regarding v_LT_ and v_IAT_ may be due to other reasons than match demands.

In this regard, it is worth mentioning that differences in endurance capacity among outfield positions (i.e., central midfielders vs. forwards and wingers) were only evident in terms of the individual thresholds (v_LT_ and v_IAT_) and not for the fixed thresholds (v_2mmol/l_ and v_4mmol/l_). This finding provides further evidence of the appropriateness of using individualized thresholds. Somewhat surprisingly, very similar performances were found for v_max_ regarding all outfield positions. This parameter was thought to best reflect the complex aerobic (below the anaerobic threshold) and anaerobic (above the anaerobic threshold) endurance demands placed upon soccer players (Dolci et al., 2020) and has been shown to largely correlate to total running distance and high-intensity distance in a German 2nd division team (Altmann et al., 2018). Nevertheless, in contrast to the submaximal lactate-based thresholds, v_max_ is highly dependent on motivation and therefore possibly less controllable, which could explain the present results.

The main strengths of this study are the broad sample size of professional soccer players competing in the 1st and 2nd German division and the use of the exact same procedures and equipment for all players. Moreover, only tests performed either at the beginning of the first half or the second half of the competitive season were analyzed. This approach ensures adequate levels of endurance performance and limits possible bias compared to pre-season testing. In addition, a number of parameters were analyzed to account for the complex demands placed upon the players' endurance capacities. The main limitation of the current study is that team tactics were not accounted for when interpreting the results, as tactics can affect the match demands of different playing positions (Baptista et al., 2019). In particular, the tactical system differed both within and between the teams the players involved in this study belonged to. This circumstance might have an impact on their endurance capacity and therefore on the results of the current study. Moreover, as the test where the highest maximum velocity was reached was used in case a player was tested more than one time, an overestimation of the endurance capacities of the players in question might be possible. In addition, previous studies provided more specific data such as percentiles for each position which might give strength and conditioning coaches additional insights and can support judging players (Schwesig et al., 2019). Nevertheless, such details are not included in the present analyses as they were not the main aim of the current study. In general, this study could have benefitted from examining the relationship between endurance capacity and match-running performance (Aquino et al., 2020). However, our study design did not allow for a reliable and comparable analysis in this regard (players belonged to different teams with different tactical formations; endurance test results were taken from different seasons and different points of time during the seasons, etc.).

Aside from that, our results suggest that outfield players of different positions possess rather similar endurance capacities. These findings could lead one to question the appropriateness of current endurance training regimes to prepare all players adequately for their positional match-running demands, which might be particularly relevant when considering wingers. Although players of these positions possess similar or even lower endurance capacities compared to other outfield playing positions, along with central midfielders, they cover both the greatest total and high-intensity distance of all positions during matches (Di Salvo et al., 2007; Bush et al., 2015). Bearing in mind that the composition of the total external load likely differs between these positions (e.g., wingers commonly cover a larger total sprinting distance than central midfielders) (Di Salvo et al., 2007; Bush et al., 2015; Dolci et al., 2020), the internal load (e.g., heart rate, lactate levels, ratings of perceived exertion) of wingers might be increased in order to account for their positional demands. In turn, when considering a longer period of time, this could yield higher levels of fatigue, and increased risk of illness and injury (Jones et al., 2017).

It is well-known that speed demands (e.g., number of sprints, total sprinting distance) during matches have dramatically increased both in the long-term (1966–2010) (Wallace and Norton, 2014) and short-term (2006–2013) (Barnes et al., 2014) with wide positions demonstrating the most pronounced increases in speed demands (Bush et al., 2015). These findings are also supported by the fact that sprint performance over various distances (linear-sprint test) increased over the recent years (Haugen et al., 2013), while endurance performance (incremental treadmill test) has remained stable (Tønnessen et al., 2013). As a consequence, it can be assumed that typical physical preparation practices might be rather speed- than endurance-focused. In turn, this could lead to coaches delivering less position-specific endurance sessions, possibly explaining the finding of this study that outfield players possess rather similar endurance capacities.

However, there might be additional reasons which might further explain the similarities of outfield players' endurance capacities in the current study. Along with previous findings by Altmann et al. (2018) that endurance capacity is related to total and high-intensity distance covered during matches, the question of the existence of player-specific match-running performance arises. Specifically, it might be worthwhile to not only investigate positional dependencies of running performance during matches but also to analyze to what extent this running performance is player specific. As such, it could be investigated if players who act in different positions rather maintain or adapt their running performance depending on the position they are instructed to play.

## Conclusion

Given the position-specific match-running demands in soccer, this study investigated the endurance capacity of soccer players according to their position by means of an incremental treadmill test. Based on a sample of 136 soccer players from the 1st and 2nd German division, the present study revealed that goalkeepers possess the lowest endurance capacity in comparison with other playing positions. While outfield players showed similar endurance performance in general, central midfielders seem to possess the highest aerobic capacity (indicated by all lactate-based thresholds) of all positions. Nevertheless, regarding the latter, the differences between outfield positions are of only small to moderate magnitude. In the context of fatigue, risk of illness, and injury, these findings could lead one to question the appropriateness of current endurance training regimes to prepare all players adequately for their positional match-running demands. However, as actual training regimes of the teams as well as risk of injury and illness were not investigated in this study, this can only be speculated. Moreover, while endurance training of players should indeed be specific to their running demands during matches, it remains unknown to what extent these demands are not only position but also player specific. As this study investigated the endurance capacities of a large sample of professional soccer players (*n* = 136) in a standardized way, the results can also serve as benchmark values for different positions.

## Data Availability Statement

The datasets for this article are not publicly available because of legal reasons. Requests to access the datasets should be directed to the corresponding author ( stefan.altmann@kit.edu).

## Ethics Statement

Ethical review and approval was not required for the study on human participants in accordance with the local legislation and institutional requirements. The patients/participants provided their written informed consent to participate in this study.

## Author Contributions

SA, RN, AW, and SH conceived the study. SA, RN, and SH performed the experiments. SA and SH analyzed data. RN, AW, and SH contributed materials and analysis tools. SA, RN, AW, and SH wrote the paper. All authors contributed to the article and approved the submitted version.

## Conflict of Interest

SH was employed by the company TSG 1899 Hoffenheim. The remaining authors declare that the research was conducted in the absence of any commercial or financial relationships that could be construed as a potential conflict of interest.

## References

[B1] Altmann S. Kuberczyk M. Ringhof S. Neumann R. Woll A. (2018). Relationships between performance test and match-related physical performance parameters. Ger. J. Exerc. Sport Res. 48, 218–227. 10.1007/s12662-018-0519-y

[B2] Aquino R. Carling C. Maia J. Vieira L. H. P. Wilson R. S. Smith N. (2020). Relationships between running demands in soccer match-play, anthropometric, and physical fitness characteristics: a systematic review. Int. J. Perform. Anal. Sport 20, 534–555. 10.1080/24748668.2020.1746555

[B3] Baptista I. Johansen D. Figueiredo P. Rebelo A. Pettersen S. A. (2019). A comparison of match-physical demands between different tactical systems: 1-4-5-1 vs 1-3-5-2. PLoS ONE 14:e0214952. 10.1371/journal.pone.0214952 30947242 PMC6448870

[B4] Barnes C. Archer D. T. Hogg B. Bush M. Bradley P. S. (2014). The evolution of physical and technical performance parameters in the English Premier League. Int. J. Sports Med. 35, 1095–1100. 10.1055/s-0034-1375695 25009969

[B5] Boone J. Vaeyens R. Steyaert A. Vanden Bossche L. Bourgois J. (2012). Physical fitness of elite Belgian soccer players by player position. J. Strength Condition. Res. 26, 2051–2057. 10.1519/JSC.0b013e318239f84f 21986697

[B6] Bush M. Barnes C. Archer D. T. Hogg B. Bradley P. S. (2015). Evolution of match performance parameters for various playing positions in the English Premier League. Hum. Mov. Sci. 39, 1–11. 10.1016/j.humov.2014.10.003 25461429

[B7] Castagna C. Impellizzeri F. M. Chaouachi A. Manzi V. (2013). Preseason variations in aerobic fitness and performance in elite-standard soccer players: a team study. J. Strength Condition. Res. 27, 2959–2965. 10.1519/JSC.0b013e31828d61a8 23442266

[B8] Cohen J. (1988). Statistical Power Analysis for the Behavioral Sciences, 2nd Edn. Hillsdale, NJ: Lawrence Erlbaum.

[B9] Di Salvo V. Baron R. Tschan H. Calderon Montero F. J. Bachl N. Pigozzi F. (2007). Performance characteristics according to playing position in elite soccer. Int. J. Sports Med. 28, 222–227. 10.1055/s-2006-924294 17024626

[B10] Dolci F. Hart N. H. Kilding A. E. Chivers P. Piggott B. Spiteri T. (2020). Physical and energetic demand of soccer. Strength Condition. J. 42, 70–77. 10.1519/SSC.0000000000000533

[B11] Faude O. Kindermann W. Meyer T. (2009). Lactate threshold concepts: how valid are they? Sports Med. 39, 469–490. 10.2165/00007256-200939060-00003 19453206

[B12] Haugen T. A. Tønnessen E. Seiler S. (2013). Anaerobic performance testing of professional soccer players 1995-2010. Int. J. Sports Physiol. Perform 8, 148–156. 10.1123/ijspp.8.2.148 22868347

[B13] Hoppe M. W. Baumgart C. Sperlich B. Ibrahim H. Jansen C. Willis S. J.. (2013). Comparison between three different endurance tests in professional soccer players. J. Strength Condition. Res. 27, 31–37. 10.1519/JSC.0b013e31824e1711 22344049

[B14] Impellizzeri F. M. Rampinini E. Marcora S. M. (2005). Physiological assessment of aerobic training in soccer. J. Sports Sci. 23, 583–592. 10.1080/02640410400021278 16195007

[B15] Jones C. M. Griffiths P. C. Mellalieu S. D. (2017). Training load and fatigue marker associations with injury and illness: a systematic review of longitudinal studies. Sports Med. 47, 943–974. 10.1007/s40279-016-0619-5 27677917 PMC5394138

[B16] Manzi V. Bovenzi A. Franco Impellizzeri M. Carminati I. Castagna C. (2013). Individual training-load and aerobic-fitness variables in premiership soccer players during the precompetitive season. J. Strength Condition. Res. 27, 631–636. 10.1519/JSC.0b013e31825dbd81 22648141

[B17] McMillan K. Helgerud J. Grant S. J. Newell J. Wilson J. Macdonald R.. (2005). Lactate threshold responses to a season of professional British youth soccer. Br. J. Sports Med. 39, 432–436. 10.1136/bjsm.2004.012260 15976165 PMC1725253

[B18] Sarmento H. Marcelino R. Anguera M. T. CampaniÇo J. Matos N. LeitÃo J. C. (2014). Match analysis in football: a systematic review. J. Sports. Sci. 32, 1831–1843. 10.1080/02640414.2014.898852 24787442

[B19] Schwesig R. Schulze S. Reinhardt L. Laudner K. G. Delank K.-S. Hermassi S. (2019). Differences in player position running velocity at lactate thresholds among male professional german soccer players. Front. Physiol. 10:886. 10.3389/fphys.2019.00886 31338041 PMC6629897

[B20] Sporis G. Jukic I. Ostojic S. M. Milanovic D. (2009). Fitness profiling in soccer: physical and physiologic characteristics of elite players. J. Strength Condition. Res. 23, 1947–1953. 10.1519/JSC.0b013e3181b3e141 19704378

[B21] Stølen T. Chamari K. Castagna C. Wisløff U. (2005). Physiology of soccer. Sports Med. 35, 501–536. 10.2165/00007256-200535060-00004 15974635

[B22] Tønnessen E. Hem E. Leirstein S. Haugen T. Seiler S. (2013). Maximal aerobic power characteristics of male professional soccer players, 1989-2012. Int. J. Sports Physiol. Perform 8, 323–329. 10.1123/ijspp.8.3.323 23118070

[B23] Wallace J. L. Norton K. I. (2014). Evolution of World Cup soccer final games 1966-2010. Game structure, speed and play patterns. J. Sci. Med. Sport 17, 223–228. 10.1016/j.jsams.2013.03.016 23643671

[B24] West J. (2018). A review of the key demands for a football goalkeeper. Int. J. Sports Sci. Coach. 13, 1215–1222. 10.1177/1747954118787493

[B25] Winter E. M. Maughan R. J. (2009). Requirements for ethics approvals. J. Sports Sci. 27, 985 10.1080/02640410903178344 19847681

[B26] Ziogas G. G. Patras K. N. Stergiou N. Georgoulis A. D. (2011). Velocity at lactate threshold and running economy must also be considered along with maximal oxygen uptake when testing elite soccer players during preseason. J. Strength Condition. Res. 25, 414–419. 10.1519/JSC.0b013e3181bac3b9 20351577

